# Down-regulation of β-lactam antibiotics resistance and biofilm formation by *Staphylococcus epidermidis* is associated with isookanin

**DOI:** 10.3389/fcimb.2023.1139796

**Published:** 2023-05-10

**Authors:** Qiang Ren, Wanhe Luo, Haoming Chi, Lili Zhang, Wei Chen

**Affiliations:** ^1^ Key Laboratory of Protection and Utilization of Biological Resources in Tarim Basin of Xinjiang Production & Construction Corps, College of Life Sciences and Technology, Tarim University, Alar, Xinjiang, China; ^2^ Engineering Laboratory for Tarim Animal Diseases Diagnosis and Control, College of Animal Science and Technology, Tarim University, Alar, Xinjiang, China

**Keywords:** *Staphylococcus epidermidis*, biofilm formation, antibiotic susceptibility, isookanin, antibiofilm activity, molecular docking

## Abstract

**Introduction:**

Biofilm formation is the major pathogenicity of *Staphylococcus epidermidis (S. epidermidis)*, which enhances bacterial resistance to antibiotics. Isookanin has potential inhibitory activity on biofilm.

**Method:**

The inhibiting mechanisms of isookanin against biofilm formation through surface hydrophobicity assay, exopolysaccharides, eDNA, gene expression analysis, microscopic visualization, and molecular docking were explored. Additionally, the combination of isookanin and β-lactam antibiotics were evaluated by the broth micro-checkerboard assay.

**Results:**

The results showed that isookanin could decrease the biofilm formation of *S. epidermidis* by ≥85% at 250 μg/mL. The exopolysaccharides, eDNA and surface hydrophobicity were reduced after treatment with isookanin. Microscopic visualization analysis showed that there were fewer bacteria on the surface of the microscopic coverslip and the bacterial cell membrane was damaged after treatment with isookanin. The down-regulation of *icaB* and up-regulation of *icaR* were observed after treatment with isookanin. Additionally, the RNAIII gene was significantly up-regulated (*p* < 0.0001) at the mRNA level. Molecular docking showed that isookanin could bind to biofilm-related proteins. This indicated that isookanin can affect biofilm formation at the initial attachment phase and the aggregation phase. The FICI index showed that the combination of isookanin and β-lactam antibiotics were synergistic and could reduce doses of antibiotics by inhibiting biofilm formation.

**Discussion:**

This study improved the antibiotic susceptibility of *S. epidermidis* through inhibition of the biofilm formation, and provided a guidance for the treatment of antibiotic resistance caused by biofilm

## Introduction

With the wide application of antibiotics in the treatment of bacterial diseases, the phenomenon of bacterial antibiotic resistance has become more serious. It has been reported that *Staphylococcus epidermidis* (*S. epidermidis*) can enhance resistance to antibiotics through the biofilm formation on the surface of infected tissues (Flemming et al., 2016). With the ability to form biofilms can provide a protective barrier for bacteria, so its exhibit higher tolerance to stress from the external environment (Xie et al., 2019; Algharib et al., 2020a). Moreover, bacteria with biofilm formation ability have higher penicillin resistance (Boonsiri et al., 2020). Therefore, the elimination of biofilm plays an important role in the treatment of disease (Dawood et al., 2022).

**Figure 1:**
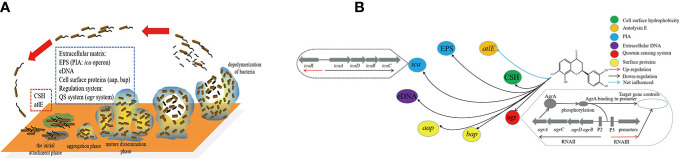
Network of transcriptional regulation of genes and material involved in *S. epidermidis* biofilm formation. **(A)** was the four phases of biofilm formation in *S. epidermidis* and the factors involved in different phases; **(B)** was the network of transcriptional regulation of isookanin inhibiting biofilm formation in *S. epidermidis*. ESP, exopolysaccharides; CSH, cell surface hydrophobicity; QS system, quorum sensing system; PIA, polysaccharide intercellular adhesin.

*S. epidermidis* biofilm formation is mainly divided into four phases (**Figure 1**). During the initial attachment phase, the autolysin E (AltE) can mediate bacterial adhesion to hydrophobic surfaces, and secretion of autolysin is disturbed by the Esp protease of *S. epidermidis* (Chen et al., 2013; Marek et al., 2021). After entering the aggregation phase, the accumulation-associated protein (Aap) can form fibrous structures on the cell surface and promote cell-to-cell interactions, and PIA-independent biofilms were mediated through Aap (Mamdoh et al., 2023). Exopolysaccharides, cell surface proteins, and extracellular DNA are the main components of *Staphylococcal* biofilm formation, and biofilm exopolysaccharides are mainly polysaccharide intercellular adhesin (PIA) (Mirzaei et al., 2019). The *ica* locus can control the production of PIA from UDP-N-acetylglucosamine (Mamdoh et al., 2023). Moreover, *Staphylococcal* accessory regulator A (SarA), biofilm-associated protein (Bap), RNA III, and QS system etc also can regulating the biofilm formation (Mamdoh et al., 2023). Because of the very complex mechanism of biofilm formation, it hinders the treatment of bacterial diseases.

Bacterial antibiotic resistance is still a serious problem, most researchers solve the problem through searching for new compounds with potent antibacterial activity, but this is not a long-term solution (Luo et al., 2022). In this concern, a new idea emerges for reducing antibiotic resistance in bacteria by inhibiting biofilm formation. It increases antibiotics susceptibility in bacteria by inhibiting biofilm, which in turn lowers the required doses for the treatment of bacterial diseases.

Flavonoids are a class of compounds with anti-biofilm activity, such as rutin, quercetin, and baicalin, etc (Mu et al., 2020; Ivanov et al., 2022; Han et al., 2023). Isookanin is also a class of flavonoids isolated and extracted from *Compositae*, which has antioxidant and anti-inflammatory effects, anti-diabetic properties, and other activities such as inhibition of α-amylase and anti-angiogenesis (Xin et al., 2021). Moreover, it has a good effect on cardiovascular diseases (Wang et al., 2015). Isookanin also could increased the relative abundance of *Bifidobacterium* and *Lactobacillus* and could reduce the primary metabolism functions of the microbiota *in vitro* (Xin et al., 2021). Isookanin has various activities, However, its inhibiting bacterial biofilms have not been reported yet.

We found that isookanin has good biological activity in inhibiting bacterial biofilm. Our objectives were to explore the effect of isookanin on the cell surface hydrophobicity, exopolysaccharides, and extracellular DNA of *S. epidermidis*, and to explore the mechanism of isookanin inhibiting biofilm using microscopic visualization and mRNA level expression. Additionally, the molecular docking was used to predict the binding of isookanin on the target protein of *S. epidermidis* (**Figure 1**). Moreover, the combination of isookanin and β-lactam antibiotics was used which enables the antibiotics to exert their bactericidal activity at lower concentrations, reducing the dosage and residues of antibiotics in animals. The emergence of antibiotic resistance was reduced, which further guides the subsequent treatment of antibiotic-resistant strains of related diseases caused by biofilms.

## Materials and methods

### Bacterial strains and growth measurements

A strong biofilm-positive strain *S. epidermidis* ATCC 35984 and antibiotics susceptibility assay quality control strains *S. aureus* ATCC 29213 were provided by Key Laboratory of Protection and Utilization of Biological Resources in Tarim Basin of Xinjiang Production & Construction Corps. Unless specified otherwise, tryptic soy agar/broth (TSA/TSB, Becton Dickinson, USA) was used to culture cells at 37 °C for 24 h. For cell growth measurements, OD value at 590nm was measured using a spectrophotometer (Bio-Rad, Winooski, USA). Each experiment was performed using at least three independent cultures.

### Assay for biofilm inhibition

The 96-well polystyrene plates were used to perform a static biofilm formation assay as previously described (Pratt and Kolter, 1998; Mu et al., 2020; Ren et al., 2020). Briefly, cells were diluted 1:100 with fresh TSB broth and cultured with different concentrations of isookanin (7.9-1000 μg/mL) for 24 h without shaking at 37 °C. Biofilms were stained with crystal violet (Sigma-Aldrich, St. Louis, MO), and dissolved in 95% ethanol (wt/vol: 0.5%). The optical density was determined at 570 nm in an enzyme-linked immunosorbent assay reader (Bio-Rad, Hercules, CA). The relative ability of biofilm formation was indicated as Relative Biofilm Formation% (RBF%) calculated by the following formula: RBF% = [Treated OD_570_/Untreated OD_570_] × 100%. Each data point was averaged from at least 12 replicate wells (four wells from each of at least three independent cultures).

### Cell surface hydrophobicity analysis

*S. epidermidis* ATCC 35984 cell surface hydrophobicity (CSH) was tested as previously described (Rosenberg et al., 1981; Mu et al., 2020). Briefly, the collected cells were washed four times through PUM buffer. PUM buffer as blank control, the 400 nm absorbance value of the bacterial solution was controlled in the range of 0.4-0.6 and recorded as OD0. OD1 was defined as the absorbance at 400 nm after n-hexadecane (Macklin, H810865) treatment. The decrease in OD value at 400 nm of the aqueous phase was taken as a measure of H%, which was calculated with the formula: H% = [(OD0-OD1)/OD0] × 100%. The experiment was repeated three times.

### Production of exopolysaccharides assay

Exopolysaccharides (EPS) in the *S. epidermidis* ATCC 35984 culture were collected as previously described (Xie et al., 2019; Mu et al., 2020). Briefly, Ethanol was used for the extraction and precipitation of the EPS of *S. epidermidis* ATCC 35984. To remove proteins, proteinase K and n-butyl alcohol were used. The aqueous layer was collected followed by dialysis with distilled water overnight. The liquid was lyophilized as an EPS sample for use.

### Quantification of eDNA

The quantity of eDNA was determined as previously described (Ivanov et al., 2022). Briefly, *S. epidermidis* ATCC 35984 cells were grown in 96-well plates containing isookanin, and without isookanin as growth control. The cells were grown at 37 °C for 24 h. Subsequently, the planktonic cells were removed, and the wells were washed with PBS. The 1 × TE buffer (Tris-EDTA buffer solution) was added to the wells and cells were mixed by pipetting. The samples were transferred to 1.5 mL tubes and centrifuged at 10,000 × g for 10 min. After removal of the supernatant, the pellet was suspended in 1 × TE buffer. The absorbance of the supernatant (260 nm) was measured after centrifugation at 10,000 × g for 15 min. We calculated the inhibition percentage of eDNA relative to the untreated control. The 1 × TE buffer was used as the control.

### Scanning electron microscopy analysis

Biofilms grown on coverslip slides were microscopically visualized as previously described (Peng et al., 2011; Mu et al., 2020). Briefly, *S. epidermidis* ATCC 35984 cells were resuspended at a density of 1.0 × 10^6^ CFU/ml in fresh TSB containing different concentrations (125-500 μg/mL) of isookanin. The Microscopic coverslip (22 × 22 mm square, Citotest, Jiang Su, China) with cells grown at 37 °C for 24 h. Cells grown in an isookanin-free medium were utilized as control and gently washed three times with PBS to remove non-adherent bacteria. Then adherent bacteria were fixed and dehydrated. The plates were fixed with 2.5% glutaraldehyde for 2 h at 4 °C. The surfaces were washed thrice with 0.01 M PBS for 15 min, and the bacteria were then dehydrated by different concentrations of ethanol (30%, 50%, 70%, 90%, 95%, and 100%) for 20 min each. After critical-point drying and coating by gold sputter, samples were examined using a scanning electron microscope (Thermo Fisher Scientific, USA).

### Confocal laser scanning microscopy analysis

*S. epidermidis* ATCC 35984 cells were cultured as SEM analysis described, and *S. epidermidis* ATCC 35984 were dyed with a Live/Dead backlight bacterial viability kit as previously described (Tan et al., 2012). Briefly, biofilm was washed three times with PBS to remove nonadherent bacteria. Then adherent bacteria were stained using the Live/Dead backlight bacterial viability kit (Molecular Probes, Eugene, OR) for 15 min at room temperature in the dark, followed by three PBS washes to remove the nonspecific stain. Samples were subsequently analyzed with a confocal laser scanning microscope (Nikon A1, Japan). The viable and nonviable cells can be distinguished under the microscope because the viable bacteria with intact cell membranes appear fluorescent green, whereas nonviable bacteria with damaged membranes appear fluorescent red.

### Gene expression analysis

To explore further the possible mechanisms for the inhibition against *S. epidermidis* biofilm by isookanin, qRT-PCR was performed to explore whether isookanin treats the transcript levels of biofilm-associated genes in *S. epidermidis* ATCC 35984 or not. Gene-specific primers were used for these genes and *gyrB* as a housekeeping control (**Table 1**). The qRT-PCR method was adapted from a previous study (Wang et al., 2011; Mu et al., 2020). QRT-PCR was performed using SYBR green PCR master mix (TransGen Biotech, Beijing, China) and ABI PRISM 7500 Real-time PCR system (RotorGene Q, Qiagen, Hilden, Germany) with two independent cultures. The 2^─△△Ct^ method was used to analyze the data of quantitative Real-Time PCR. The experiment was repeated three times.

**Table 1:** Biofilm-related gene primers were used for quantitative reverse transcriptase-PCR.

Gene	Primer sequences(5’-3’)
*aap* F	GCAGGCATGCTTAATAAGGAC
*aap* R	AGAACCTACAACTTCAGAACC
*icaR* F	CATTGACGGACTTTACCAGTTTT
*icaR* R	ATCCAAAGCGATGTGCGTAG
*icaB* F	GAAACAGGCTTATGGGACTTTG
*icaB* R	CAAGTGCGCGTTCATTTTT
RNAIII F	ACTAAATCACCGATTGTAGAAATGATATCT
RNAIII R	ATTTGCTTAATCTAGTCGAGTGAATGTTA
*atlE* F	GATGGATTGCTGCTAAGGATTT
*atlE* R	TATCGGTTTGCTTTTGTTGG
*gyrB* F	TGACGAGGCATTAGCAGGTT
*gyrB* R	GTGAAGACCGCCAGATACTTT

### Molecular docking

To explore the prediction of drug targets of isookanin on biofilm formation-related proteins of *S. epidermidis*. The PDB file for IcaR, Aap A-domain, Aap B-domain, Esp, Bap B domain, and Bap C region was downloaded from the RCSB database (https://www.pdbus.org/) as the receptor protein, the PDB ID 2ZCN, 7SIE, 5TU8, 4JCN, 7C7R, and 7DM0, respectively; the isookanin molecular structure was downloaded from the PubChem database (https://pubchem.ncbi.nlm.nih.gov/) for energy minimization to generate a structure file that could be adapted for molecular docking. The receptor protein and isookanin were processed by AutoDockTools 1.5.6 software. Subsequently, the appropriate scores and conformation results were selected and visualized using PyMOL (https://pymol.org/2/) and Discovery Studio 2020 (Kang et al., 2022).

### MIC assay

The MICs of antibiotics against *S. epidermidis* ATCC 35984 were measured by 96-well polystyrene plates as previously reported with slight modifications (Cole et al., 1997; Beckloff et al., 2007). The administrated antibiotics were penicillin (Biotopped, Beijing, China), ceftiofur sodium (Dr. Ehrenstorfer, LGC, Germany), cefquinome sulfate (Dr. Ehrenstorfer, LGC, Germany), cloxacillin sodium (Dr. Ehrenstorfer, LGC, Germany), and oxacillin sodium (Dr. Ehrenstorfer, LGC, Germany), respectively. Briefly, cells were diluted 1: 100 with fresh TSB broth and cultured with different concentrations of antibiotics (0.25-256 μg/mL) for 24 h without shaking at 37 °C. The wells containing only bacterial liquid and non-antibiotics were used as positive controls, and the wells containing only culture medium without bacteria were used as blank controls. These assays were performed in triplicates including biological duplicates. *S. aureus* ATCC 29213 was used as a quality-control strain.

### Broth Micro-Checkerboard assay

Broth Micro-Checkerboard was used for isookanin and five β-lactam antibiotics combination as previously reported with slight modifications (Rogers et al., 2010; Yan et al., 2022). Briefly, 50 μL of ploidy dilution antibiotics working solution add it to columns 2-12 of the 96-well plate, and 50 μL of ploidy dilution isookanin solution added it to row A-G of the plate. Except for A12, 1.0 × 10^6^ CFU/mL of 100 μL bacterial suspension was then added to each well. Both blank (A12 plate) and positive (H1 plate) controls were set up. Subsequently, the plates were incubated at 37 °C for 24 h. Finally, the results were observed. The fractional inhibition concentration index (FICI) was calculated using the following formula: FICI = CA _combination_/CA _single_ + CB _combination_/CB _single_. CA _combination_ and CB _combination_ represent isookanin and antibiotics concentration in combination. CA _single_ and CB _single_ represent isookanin or antibiotics were used alone concentration. Where synergy was defined as a FICI of ≤ 0.5, the additive was defined as a FICI of > 0.5 but < 1, indifference was defined as a FICI of ≥ 1 but < 4, while antagonism was defined as a FICI of ≥ 4. The experiment was repeated three times.

### Statistical analysis

All the experimental data were processed by GraphPad Prism 7 (GraphPad Software, San Diego, CA, USA) and a t-test was used for statistical analyses. A *P* value of < 0.05 was considered statistically significant.

## Results

### Minimum inhibitory concentration and minimum biofilm Inhibitory concentration

**Figure 2:**
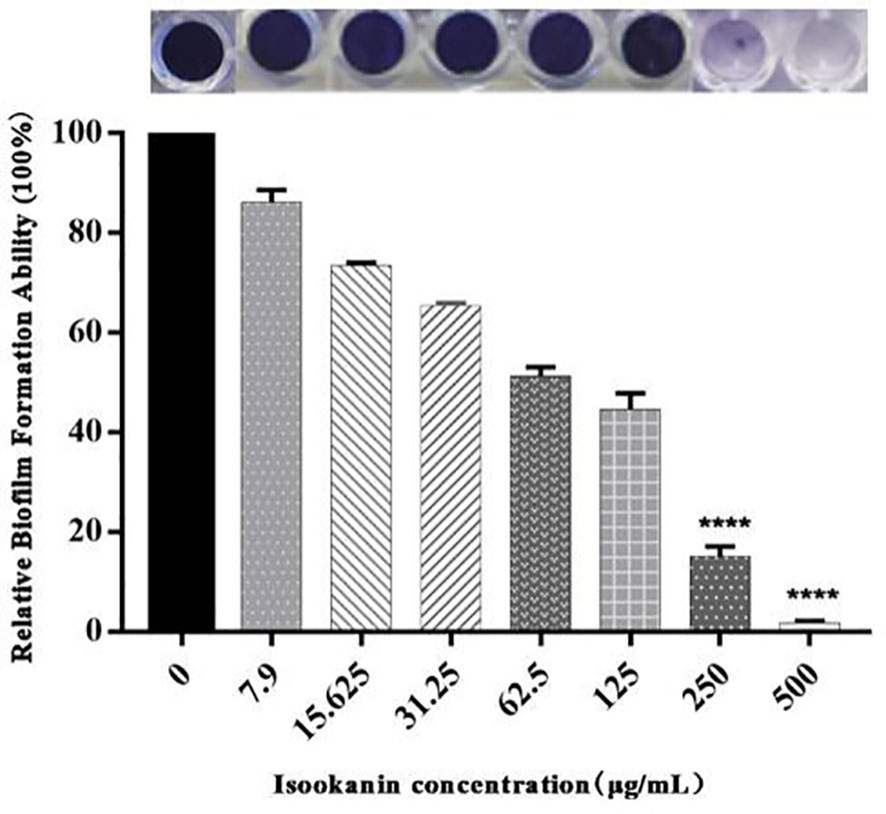
Relative biofilm formation of different isookanin concentrations against *S. epidermidis* ATCC 35984. The bacteria without isookanin treatment were used as the growth control. Biofilm formation of *S. epidermidis* ATCC 35984 treated with 250 μg/mL and 500 μg/mL was significantly inhibited compared with that of bacteria without isookanin treatment (****, *P* < 0. 0001).

In this study, the MICs of penicillin, ceftiofur sodium, cefquinome sulfate, cloxacillin sodium, and oxacillin sodium against *S. epidermidis* ATCC 35984 were 16, 4, 2, 8, and 8 μg/mL, respectively (**Table 2**). On the other hand, the MBIC of isookanin against *S. epidermidis* ATCC 35984 was 250 μg/mL. Specifically, the biofilm formation of *S. epidermidis* ATCC 35984 decreased by ≥ 85% at 250 μg/mL and by ≥ 95% at 500 μg/mL. Biofilm formation of *S. epidermidis* ATCC 35984 treated with 250 and 500 μg/mL isookanin was significantly inhibited compared with that of bacteria without isookanin treatment (*P* < 0. 0001) (**Figure 2**).

**Table 2:** The MIC and MBIC of different medicines against *S. epidermidis*.

Strains	Antibiotics (MIC)					Anti-biofilm medicines
	Penicillin	Ceftiofur sodium	Cefquinome sulfate	Cloxacillin sodium	Oxacillin sodium	Isookanin
*S.epidermidis* ATCC 35894	16	4	2	8	8	250

### Isookanin decreased cell surface hydrophobicity in *S. epidermidis*

**Figure 3:**
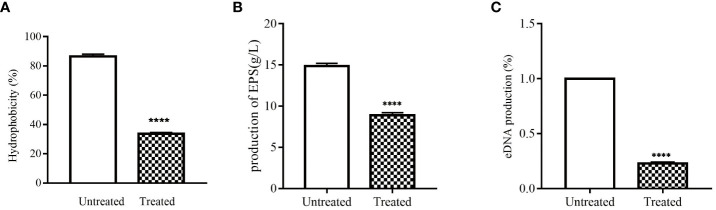
Effect of isookanin on the cell surfaces and extracellular matrix of *S. epidermidis* ATCC 35984. **(A)** was the effect of isookanin on the hydrophobicities of cell surfaces; **(B)** was the effect of isookanin on the production of EPS; **(C)** was the effect of isookanin on the production of eDNA. The data were representative of the results from three independent experiments. *S. epidermidis* ATCC 35984 treated with 250 μg/mL was significantly inhibited compared with that of bacteria without isookanin treatment (****, *P* < 0. 0001).

The cell surface hydrophobicity can enhance the adhesion of *S. epidermidis*, thereby promoting the formation of biofilm. Therefore, decreasing cell surface hydrophobicity can further explore the mechanism of isookanin inhibiting the biofilm formation of *S. epidermidis*. Isookanin can significantly reduce the cell surface hydrophobicity, thus reducing the adhesion of *S. epidermidis* ATCC 35984 (*p* < 0.0001) (**Figures 3A**, **1B**).

### Isookanin reduced the production of EPS in *S. epidermidis*

In this study, EPS in *S. epidermidis* ATCC 35984 was obtained by alcohol precipitation and dialysis. By weighing, it was found that the production of EPS in *S. epidermidis* ATCC 35984 was changed after treatment with isookanin. The production of EPS in the untreated group was 14.85 g/L, and after treatment with 250 μg/mL of isookanin, the production of EPS was reduced to 8.88 g/L (**Figures 3B**, **1B**), and the production of EPS was reduced 1.62 times after treatment with isookanin.

### Isookanin decreased eDNA production in *S. epidermidis*

Biofilm can increase antibiotic resistance due to the enabling of the horizontal transfer of antibiotic resistance genes though eDNA (Ivanov et al., 2022). Isookanin can decreased the production of eDNA from *S. epidermidis* ATCC 35984. Compared with the untreated group, the production of eDNA after treatment with isookanin was only 22.4% (*p* < 0.0001) (**Figures 3C**, **1B**).

### Scanning electron microscopy analysis

**Figure 4:**
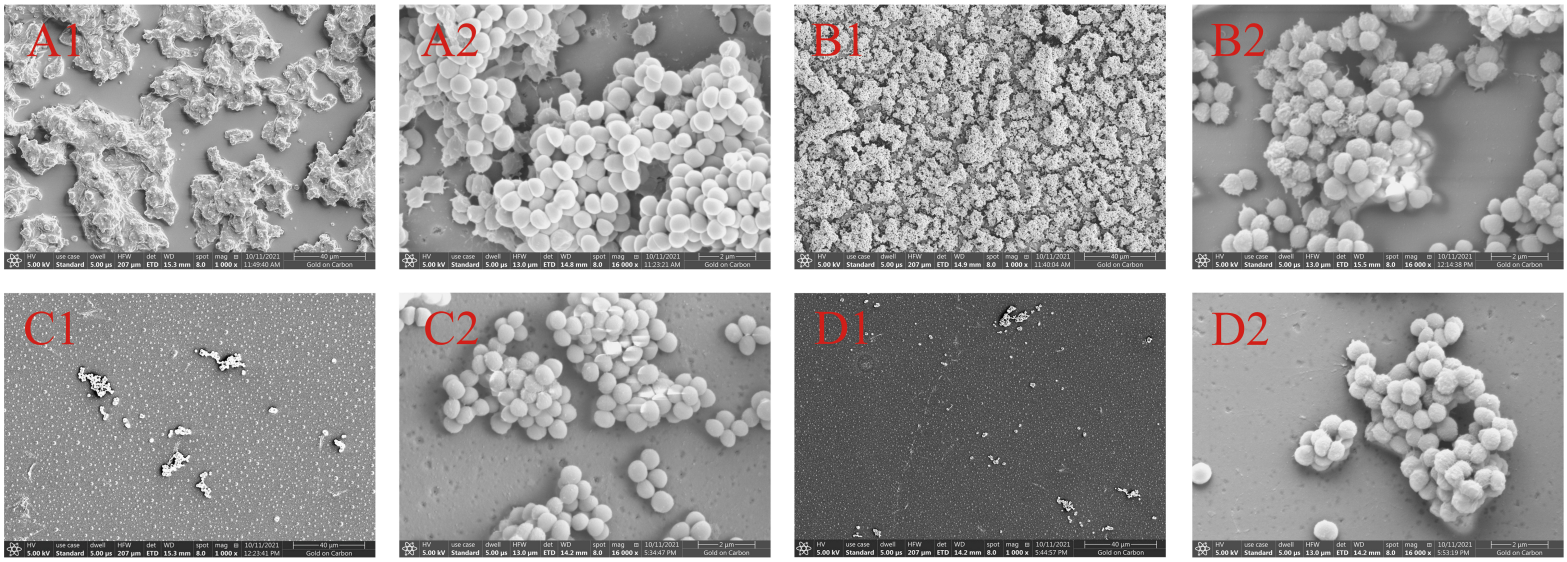
Scanning electron micrographs (SEM) of biofilms formed by *S. epidermidis* ATCC 35984. *S. epidermidis* ATCC 35984 was treated with 0 μg/mL **(A)**, 125 μg/mL **(B)**, 250 μg/mL **(C)**, and 500 μg/mL **(D)** by isookanin. 1 and 2 as defined Magnification, × 1000 and × 16000, respectively.

The biofilm formation of *S. epidermidis* ATCC 35984 was observed by scanning electron microscopy. Without treatment, the surface of *S. epidermidis* ATCC 35984 has some protrusions and was not smooth. Additionally, the *S. epidermidis* ATCC 35984 cells gather together, and a large number of cells were observed in coverslip surface (**Figures 4A1, A2**). When treated with 125 μg/mL isookanin, the growth of the *S. epidermidis* cells was not inhibited, and secreted substances were produced (**Figures 4B1, B2**). When treated with 250 μg/mL isookanin, the distribution of the *S. epidermidis* cells was sparse; the *S. epidermidis* grew into isolated individual colonies (**Figure 4C1**). The surface of *S. epidermidis* ATCC 35984 was smooth and fewer secretions were produced (**Figure 4C2**). Further, when the concentration of isookanin was increased to 500 μg/mL, only a few bacterial microcolonies were observed, and no biofilm was formed on the surface of *S. epidermidis* ATCC 35984 (**Figures 4D1, D2**).

### Isookanin interfered with cell wall and membrane integrity

**Figure 5:**
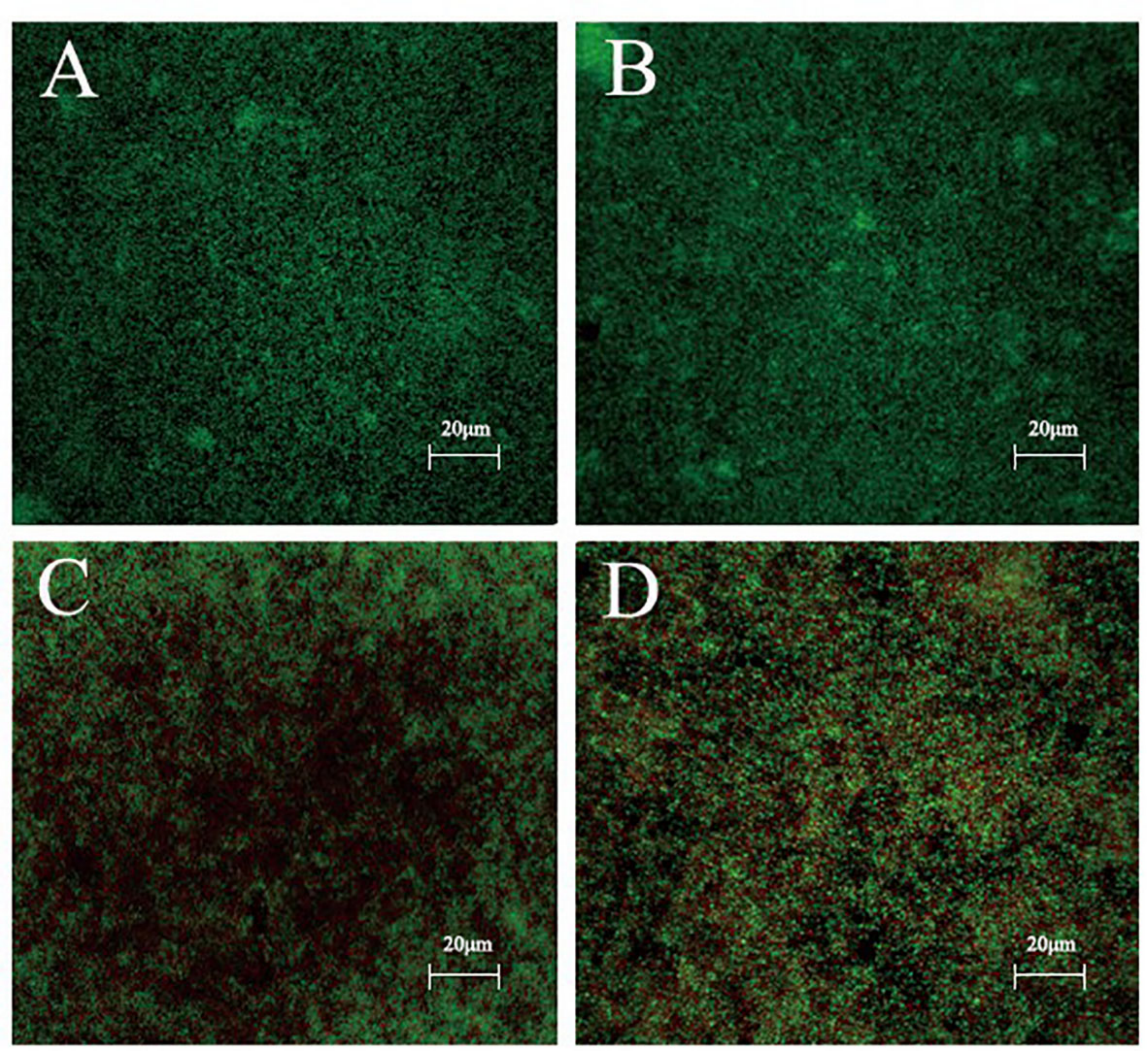
Confocal laser scanning microscopy (CLSM) analysis of bacterial viability in a biofilm on a coverslip surface. *S. epidermidis* ATCC 35984 was incubated in TSB with 0 μg/mL **(A)**, 125 μg/mL **(B)**, 250 μg/mL **(C)** and 500 μg/mL **(D)** isookanin treatment. Bacteria were stained with green fluorescent SYTO 9 and red fluorescent propidium iodide, resulting in live cells appearing green and dead cells appearing red under CLSM. Magnification, × 400.

After staining with the Live/Dead backlight bacterial viability kit, the biofilm formation stability of *S. epidermidis* ATCC 35984 was analyzed by CLSM. Without and/or with low isookanin concentration (125 μg/mL) treatment, the *S. epidermidis* cells had intact cell membranes, so green fluorescence appeared (**Figures 5A, B**). When treated with 250 μg/mL isookanin, red fluorescence appeared, which indicates that the cell membrane of *S. epidermidis* was damaged, and red fluorescence enters the cell for staining (**Figure 5C**). Evidently, when treated with 500 μg/mL isookanin, more red fluorescence has appeared (**Figure 5D**).

### Isookanin regulates multiple biofilm-related genes to inhibit biofilm formation

**Figure 6:**
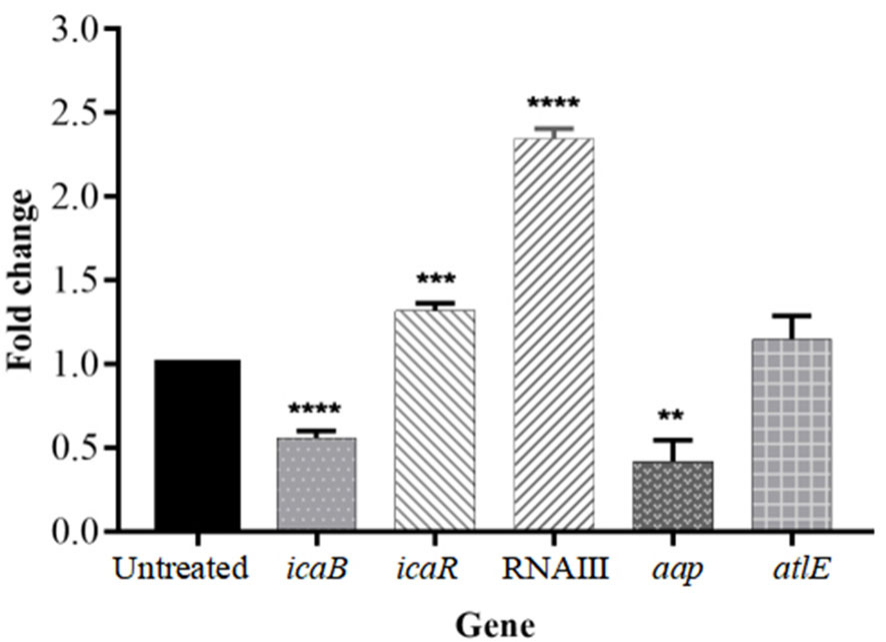
Relative mRNA expression of biofilm-related genes in *S. epidermidis* with or without the treatment of isookanin (250 μg/mL). Expression of *gyrB* was used as a housekeeping control. The data are representative of results from three independent experiments. The asterisks represent statistical significance **, *P* < 0.01, ***, *P* < 0.001, and ****, *P* < 0.0001.

The formation of biofilm was affected by a variety of regulatory factors. To gain further insight into the molecular basis of biofilm inhibition by isookanin, the expression of biofilm-related genes was analyzed including the *ica* locus (*icaR* and *icaB*), *aap*, and *altE* by qRT-PCR, and the RNAIII negatively regulates *agr* of the QS system (Kuehl et al., 2009), the RNAIII also was analyzed at the RNA expression level. In the biofilm cells, the exposure of isookanin resulted in the down-regulation of repressor *icaB* (*p* < 0.0001), while an up-regulation of *icaR* was reported (*p* < 0.001) (**Figure 6**). Additionally, RNAIII was significantly up-regulated (*p* < 0.0001) (**Figure 6**), and *aap* gene was down-regulated (*p* < 0.01) (**Figure 6**). On the other hand, autolysin E (*altE*) was expressed at the mRNA level. The isookanin treatment had no significant effect on the mRNA level of the *altE* gene, and the *altE* gene was constant at the mRNA level (**Figures 6**, **1B**).

### Isookanin inhibits the biofilm formation of *S. epidermidis* by binding to the target protein

**Figure 7:**
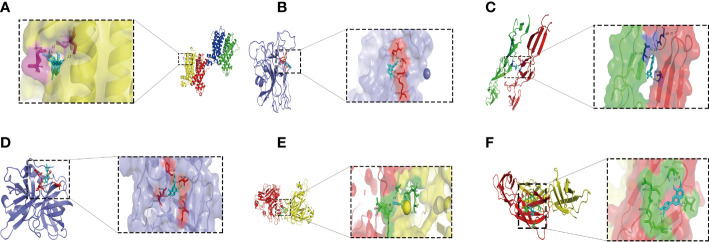
The molecular docking results of the IcaR, Aap A-domain, Aap B-domain, Esp, Bap B domain, Bap C region and isookanin. Light blue was isookanin, yellow dotted line was hydrogen bond, the places where hydrogen bonds and amino acid residues combine were marked with different colors. **(A)** was the molecular docking diagram of isookanin and IcaR (2ZCN); **(B)** was the molecular docking diagram of isookanin and Aap A-domain (7SIE); **(C)** was the molecular docking diagram of isookanin and Aap B-domain (5TU8); **(D)** was the molecular docking diagram of isookanin and ESP (4JCN); **(E)** was the molecular docking diagram of isookanin and Bap B domain (7C7R); **(F)** was the molecular docking diagram of isookanin and Bap C region (7DM0).

To determine the target protein and the optimal binding site of isookanin and *S. epidermidis*, the surface binding cavity of IcaR, Aap A-domain, Aap B-domain, Esp, Bap B domain, and Bap C region was predicted and analyzed based on the surface rolling ball model. The analysis revealed they can provide a binding site for isookanin. The results of the docking studies showed that the isookanin molecule form IcaR, Aap A-domain, Aap B-domain, Esp, Bap B domain, and Bap C region H-bonds and amino acid residues were three (ASP 68, GLY 75, GLU 123), four (LYS 359, ARG 418, ASP 596, GLY 599), three (ASP 87, ASN 177, GLU 186), five (ASP 86, ALA 87, PRO 180, THR 182, ASN 244), six (GLN 612, ASP 614, LYS 616, ASN 629, ASN 650, LYS 651 and five (ASN 120, ASP 121, THR 123, THR 127, THR 130), respectively (**Table 3** and **Figure 7**). The binding free energy of -6.19, -7.63, -5.45, -7.58, -7.82, and -6.6 Kcal/mol, respectively (**Table 3** and **Figure 7**).

**Table 3:** Docking results of isookanin, the IcaR (ID: 2ZCN), the Aap A-domain (ID: 7SIE), the Aap B-domain (ID: 5TU8), the Esp (ID: 4JCN), the Bap B domain (ID: 7C7R), and the Bap C region (ID: 7DM0).

	Isookanin					
	IcaR	Aap A domain	Aap B domain	ESP	Bap B domain	Bap C region
Binding-energy (Kcal/mol)	-6.19	-7.63	-5.45	-7.58	-7.82	-6.6
H-bonds (n)	3	4	3	5	6	5
Binding site	ASP 68 GLY 75 GLU 123	LYS 359 ARG 418 ASP 596 GLY 599	ASP 87 ASN 177 GLU 186	ASP 86 ALA 87 PRO 180 THR 182 ASN 244	GLN 612 ASP 614 LYS 616 ASN 629 ASN 650 LYS 651	ASN 120 ASP 121 THR 123 THR 127 THR 130

ASP, Aspartic acid; GLY , Glycine; GLU , Glutamic acid; LYS , Lycine; ARG , Arginine; ASN, Asparagine; ALA , Alanine; PRO , Proline; THR , Threonine; GLN , Glutamine.

### Interactions between isookanin and antimicrobial compounds (FICI index)

In order to evaluate the interaction between isookanin and β-lactam antibiotics, the FICI values of isookanin combined with five different antibiotics (penicillin, ceftiofur sodium, cefquinome sulfate, cloxacillin sodium, and oxacillin sodium) against *S. epidermidis* ATCC 35984 were determined. In this study, the FICI of isookanin combined with penicillin, ceftiofur sodium, cefquinome sulfate, cloxacillin sodium, and oxacillin sodium against *S. epidermidis* ATCC 35984 were 0.3125, 0.25, 0.375, 0.375, and 0.25, respectively. Thus, the antibacterial effect of combining isookanin and five different β-lactam antibiotics on *S. epidermidis* ATCC 35984 showed synergistic effects, and the concentrations of isookanin and five different antibiotics (penicillin, ceftiofur sodium, and cefquinome sulfate, cloxacillin sodium, and oxacillin sodium) in combinations were 15.625 and 4 μg/mL, 31.25 and 0.5 μg/mL, 31.25 and 0.5 μg/mL, 31.25 and 2 μg/mL, and 31.25 and 1 μg/mL, respectively (**Table 4**).

**Table 4:** Antibacterial activity of isookanin combined with antibiotics against *S. epidermidis* ATCC 35984 *in vitro* (FICI index).

Medicines combination	Antimicrobial MIC (μg/mL)	Compound MBIC (μg/mL)	Combined dose (μg/mL)	FICI	Compoundtype
Penicillin+Isookanin	16	250	4/15.625	0.3125	synergy
Ceftiofur sodium+Isookanin	4	250	0.5/31.25	0.25	synergy
Cefquuinome sulfate+Isookanin	2	250	0.5/31.25	0.375	synergy
Cloxacillin sodium+Isookanin	8	250	2/31.25	0.375	synergy
Oxacillin sodium+Isookanin	8	250	1/31.25	0.25	synergy

## Discussion

Biofilm formation is a challenge to conventional antibiotics for treating *Staphylococcus*-related diseases. Many conventional antibiotics exhibit high antibiotic resistance to *Staphylococcus*, there is an ever-increasing amount of research aimed at increasing the antibiotic susceptibility of bacteria by inhibiting biofilm formation (Tan et al., 2012). More and more anti-biofilm medicines have been evaluated by researchers (Peng et al., 2011; Mu et al., 2020 **;** Kang et al., 2022).

Isookanin is a class of flavonoids isolated and extracted from *Compositae*, which has antioxidant and anti-inflammatory, anti-diabetic properties, inhibition α-amylase of anti-angiogenesis and treating cardiovascular diseases (Wang et al., 2015; Xie et al., 2019). However, there is no relevant reported on the inhibition of biofilm formation and its inhibition mechanism. As a compound isolated from *Compositae*, isookanin is low toxic and harmless. However, it has no relevant bacteriostatic activity, and it can inhibit biofilm formation. The inhibition rate of 250 μg/mL isookanin for biofilm formation was higher than 85%. Besides, the more the concentration increased, the more the inhibition phenomenon was obvious and concentration-dependent. Our results were consistent with other previous reports that many anti-biofilm medicines are concentration-dependent (Peng et al., 2011; Mu et al., 2020).

A combination of antibiotics can effectively reduce the dose of the two antibiotics, which in turn can achieve the purpose of bacteriostasis at a lower concentration (Algharib et al., 2020b; Hussein et al., 2022; Yan et al., 2022). Our study successfully achieved the goal of improving antibiotic susceptibility by inhibiting biofilm formation. The checkerboard method defines a synergistic effect of the combination of isookanin and five β-lactam antibiotics. The dosages of conventional antibiotics, as well as the residue of antibiotics were reduced in animals. Eventually, the phenomenon of bacterial resistance was alleviated. The combination of ceftiofur sodium, cefquinome sulfate, penicillin, cloxacillin sodium, and oxacillin sodium was used successfully with doses of 0.5, 0.5, 4, 2, and 1 μg/mL, respectively. *S. epidermidis* is inhibited by 1/4-1/8 of the antibiotic concentration alone and improves the antibiotic susceptibility of *S. epidermidis*.

This study explored the effect of isookanin on the composition of EPS and surface hydrophobicity of *S. epidermidis* to disclose the inhibiting biofilm formation mechanisms. EPS is one of the key factors, which is responsible for biofilm formation by *S. epidermidis* (Xie et al., 2019). In this study, isookanin treatment reduced *S. epidermidis* EPS production by 1.67-fold (**Figure 2B**, *p* < 0.0001), thereby inhibiting biofilm formation. Actinomycin D has also been reported to reduce EPS production (Mu et al., 2020). Isookanin also reduced *S. epidermidis* cell surface hydrophobicity from 85.2% to 33.6% (**Figure 2A**, *p* < 0.0001). Generally, *S. epidermidis* with more prefers to grow on hydrophobic material surfaces (Dunne, 2002). Additionally, cell surface hydrophobicity helps bacteria adhere to hydrophobic surfaces, and the reduced hydrophobicity makes cells less likely to grow on hydrophobic surfaces, thereby inhibiting biofilm formation (Xie et al., 2019). This is an effective way to inhibit *staphylococcal* biofilm formation by reducing cell surface hydrophobicity, which is the most common cause of recurrent infections (Lee et al., 2016). Isookanin can decrease the production of eDNA, this is consistent with the reported effects of other flavonoids (Ivanov et al., 2022; Tao et al., 2022). The eDNA is one of the components of the extracellular matrix, and the reduction of eDNA production inhibited bacterial proliferation. Additionally, eDNA production is also related to QS and cell surface hydrophobicity. Thus, reducing its production can inhibit the biofilm formation (Ivanov et al., 2022).

To further evaluate the effect of isookanin on biofilm formation, the changes in biofilms after isookanin treatment were observed by microscopic visualization. SEM and CLSM revealed that biofilm formation and stability were inhibited only at high concentrations of 250 μg/mL and 500 μg/mL isookanin. What’s more, the biofilm formation was not inhibited after isookanin treatment below MBIC (250 μg/mL). These results indicated that isookanin had the potential to block bacterial colonization and biofilm formation. Moreover, with the increase of isookanin concentration, the inhibition effect of biofilm formation of *S. epidermidis* ATCC 35984 was more obvious. This indicated that isookanin against biofilm formation of *S. epidermidis* ATCC 35984 had concentration-dependent.

The formation of biofilm was affected by a variety of regulatory factors, the PIA appears to play a critical role in the *S. epidermidis* biofilm formation, which is responsible for intercellular adhesion (Olson et al., 2006). The PIA is produced by glucuronyl transferases encoded by the *ica* operon which comprises four intercellular adhesion genes (*icaA*, *icaB*, *icaC*, and *icaD*). It was negatively regulated by *icaR*, encoding a transcriptional repressor of icaADBC operon (Nuryastuti et al., 2009). The *icaR* repression of *ica* operon transcription contributes to reduced production of PIA and causes an impaired biofilm-producing ability (Conlon et al., 2002). Our results showed a down-regulation of *icaB* and an up-regulation of *icaR*. The PIA is a type of EPS produced by *S. epidermidis.* Therefore, we examined EPS production and found a decreased production of EPS in treated groups (**Figures 2B**, **1**). This was consistent with previous results (Mu et al., 2020). AltE plays an important role in microbial surface components recognizing adhesive matrix molecules to bind to the hydrophobic surface and complete the initial attachment phase (Marek et al., 2021). The *altE* gene was constant at the mRNA level and the accumulation associated protein (*aap*) gene was significantly down-regulated with isookanin treatment (**Figure 6**, **1**, *P* < 0.01). This indicates that isookanin affects biofilm formation in both the initial attachment phase and the aggregation phase in *S. epidermidis*, which was inconsistent with previous research (Mu et al., 2020). The RNAIII can negatively regulate *agr* of the QS system (Kuehl et al., 2009), RNAIII gene was significantly up-regulated at the mRNA level in our research. This suggested that isookanin can inhibit biofilm formation by inhibiting QS.

Isookanin could bind to the biofilm-related proteins (IcaR, Aap, Bap, and Esp) by forming hydrogen bonds, and hydrogen bonds can maintain the stability of the isookanin-proteins complex. To date, no studies have been reported on whether isookanin could bind to biofilm-related proteins. Other studies have shown that gemifloxacin can bind to TcaR protein related to the biofilm formation of *S. epidermidis* by molecular docking (Vuppala et al., 2022). Quercetin (a kinds of flavonoids) can bind an essential protein called ClfB in forming biofilm in *S. aureus*, and it can inhibits *S. aureus* infection by amino acid residues and hydrogen bonds (Kang et al., 2022). Isookanin and Aap A domain (-7.63 Kcal/mol), Esp (-7.58 Kcal/mol), and Bap B domain (-7.82 Kcal/mol) have higher binding energy, and they can bind IcaR protein (-6.19 Kcal/mol). Thus, isookanin can up-regulate *icaR* genes and inhibit the production of PIA in *S. epidermidis*, thereby inhibiting the biofilm formation.

## Conclusion

In conclusion, our study indicated that isookanin exhibited biofilm formation inhibition *via* decreasing PIA production, eDNA production and cell surface hydrophobicity. Preliminary exploration of the inhibitory mechanism of isookanin on the biofilm formation of *S. epidermidis* found that isookanin has inhibitory effects on the initial attachment phase and the aggregation phase of biofilm formation (**Figure 1**). Also, the combination of isookanin and five β-lactam antibiotics greatly reduces the dosage of antibiotics. This achieves the expected purpose of improving the antibiotic susceptibility of *S. epidermidis* through inhibition of the biofilm formation. The results of this study provided some advice for the treatment of β-lactam resistance *S. epidermidis* infection caused by biofilm.

## Data availability statement

The datasets presented in this study can be found in online repositories. The names of the repository/repositories and accession number(s) can be found in the article/supplementary material.

## Author contributions

Methodology: QR and WL. Figure modification: QR and HC. Writing-original draft preparation: QR and WL. Writing-review and editing: LZ and WC. Funding acquisition: WC and WL. All authors have read and agreed to the published version of the manuscript. All authors contributed to the article.
